# Association of the “Weekend Warrior” and Other Physical Activity Patterns with Metabolic Syndrome in the South Korean Population

**DOI:** 10.3390/ijerph192013434

**Published:** 2022-10-18

**Authors:** Yun Seo Jang, Hye Jin Joo, Yun Hwa Jung, Eun-Cheol Park, Suk-Yong Jang

**Affiliations:** 1 Department of Public Health, Graduate School, Yonsei University, Seoul 03722, Korea; 2 Institute of Health Services Research, Yonsei University, Seoul 03722, Korea; 3 Department of Preventive Medicine, College of Medicine, Yonsei University, Seoul 03722, Korea; 4 Department of Healthcare Management, Graduate School of Public Health, Yonsei University, Seoul 03722, Korea

**Keywords:** KNHANES, metabolic syndrome, moderate-vigorous physical activity, physical activity intensity, regularly active, weekend warrior

## Abstract

These days, it is not common for people to have time to do physical activities regularly because of their own work. So, they perform physical activities all at once, which is often called the “weekend warrior”. Therefore, this study aimed to examine the association of the “weekend warrior” and other physical activity patterns with metabolic syndrome. Data from the Korea National Health and Nutrition Examination Survey were used, and 27,788 participants were included. The participants were divided into inactive, weekend warriors, and regularly active based on physical activity patterns. The risk of metabolic syndrome in each group was analyzed using multiple logistic regression. The inactive and weekend warrior groups showed a higher likelihood of developing metabolic syndrome than the regularly active groups (weekend warrior: odds ratio (OR) 1.29, confidence interval (CI) 1.02–1.65; inactive: OR 1.38, CI 1.25–1.53). According to the physical activity patterns, the weekend warrior group showed a dose-response relationship compared to the regularly active group (only moderate: OR 1.85, CI 1.25–2.72; only vigorous: OR 1.41, CI 0.93–2.14; both: OR 0.84, CI 0.56–1.27). This study found increasing the amount of physical activity and performing vigorous-intensity physical activity helped manage metabolic syndrome in the weekend warrior group.

## 1. Introduction

Physical activity can prevent obesity by improving health and reducing the risk of cardiovascular diseases and cancer by controlling chronic diseases such as hypertension and diabetes [1,2,3,4]. Especially, increased physical activity has been associated with biomarkers of endothelial dysfunction in (pre)diabetes than in normal glucose metabolism [5,6]. In addition, the combination of physical activity and dietary habits produce synergistic effects not only in the prevention and treatment of obesity at all ages, but also in the health status of certain patients [7]. Since these physical activities ultimately contribute to improving quality of life, prolonging healthy lifespan, and reducing the burden of individual medical expenses, many countries encourage people to follow physical activity guidelines [8,9]. According to the World Health Organization (WHO) 2020 guidelines on physical activity and sedentary behavior [10], adults are recommended to perform 150 to 300 (min/week) of moderate-intensity physical activity, 75 to 150 (min/week) of vigorous-intensity physical activity, or an equivalent combination of two intensity physical activities. Considering that performing physical activity only on weekends or part-time may be a more convenient option, a previous study has shown that it is important to achieve the recommended level of physical activity, regardless of the number of exercise sessions per week [11]. However, it is unclear whether performing the same amount of physical activity consistently over many days and concentrating these activities into fewer days has the same benefits.

The prevalence of metabolic syndrome has been on the rise in the last 12 years, especially in men [12]. According to the Korean Society of the cardiometabolic syndrome in 2018, about 23% of adults have metabolic syndrome, and the prevalence when separated by sex is 27.9% for males and 17.9% for females. In addition, the prevalence of the metabolic syndrome is 45.3% high in the elderly over the age of 65. Metabolic syndrome refers to a condition wherein hypertension, hyperglycemia, dyslipidemia, and abdominal obesity occur simultaneously [13], which causes insulin resistance and obesity, and increases cardiovascular disease risk factors [14,15,16]. The WHO has announced that the risk factors for metabolic syndrome contribute to increasing the risk of chronic diseases. To combat this challenge effectively, the National Cholesterol Education Program (NCEP) Adult Treatment Panel III suggested that weight control, increased physical activity, and diet-related lifestyle changes are essential [17].

Meta-analysis results of studies on physical activity and metabolic syndrome advocate vigorous-intensity physical activity that substantially exceeds the level recommended by the WHO [18,19]. It also suggests that a mix of moderate and vigorous-intensity physical activity, rather than an inactive group, can provide similar benefits in preventing metabolic syndrome [19]. However, the minimum weekly frequency and amount of physical activity for individuals with no time to spare, which are associated with metabolic syndrome, remain unknown. There are studies on disease-related mortality between those who focus on physical activity once or twice a week (i.e., weekend warrior) and those who exercise multiple days over a week (i.e., regularly active) [20,21]; however, there are only a few studies examining the prevalence of disease between the two groups.

This study aimed to examine the association between physical activity patterns and metabolic syndrome and to determine whether the type of physical activity and time to exercise for a week in the weekend warrior group have a potential relationship with the prevalence of metabolic syndrome.

## 2. Methods

### 2.1. Data

The Korea National Health and Nutrition Examination Survey (KNHANES) cross-sectional data from 2016 to 2020 were used in this study. The KNHANES was conducted by the Korea Centers for Disease Control and Prevention (KDCA) since 1998. The KNHANES provides reliable data for evaluating and developing health policies and programs in Korea. This survey includes each year as a survey sample and collects information on socioeconomic status, quality of life, health-related behaviors, healthcare utilization, anthropometric measures, biochemical and clinical profiles for non-communicable diseases [22]. It provides a representative sample of the South Korean population using a stratified, multi-stage, cluster sampling design based on sex, age, and geographic area. This study did not require approval from the ethics committee because it used publicly accessible data in compliance with the Declaration of Helsinki.

### 2.2. Participants

**Figure 1:**
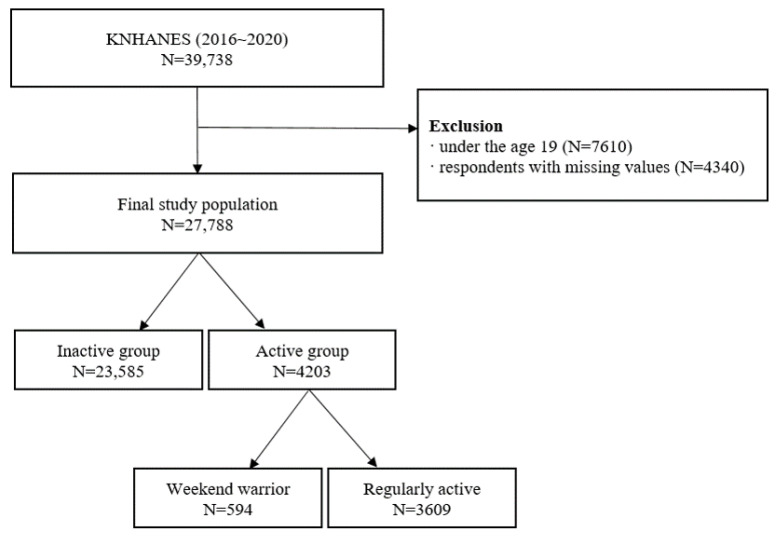
Flowchart of the study participants displaying the inclusion and exclusion.

In total, 39,738 participants were enrolled in the KNHANES from 2016 to 2020. Among them, individuals younger than 19 years of age were excluded due to a lack of data on physical activity (N = 7610). Participants with missing data on metabolic syndrome and physical activity patterns were also excluded from the list of eligible participants (N = 4143). A total of 27,788 participants were finally considered for analysis, excluding 197 participants with missing covariate data (Figure 1).

### 2.3. Variables

The primary variable in this study was the physical activity pattern. Physical activity patterns were assessed using the following six questions: (1) Do you usually perform physical activities of vigorous intensity where your heart beats very fast or you are out of breath for at least 10 min, except for work and location movement?”, (2) “In a week, how many days do you usually perform physical activities of vigorous intensity like above?”, (3) “How long do you usually perform physical activities of vigorous intensity like above?”, (4) “Do you usually perform physical activities of light-to-moderate intensity where your heart beats a little fast or experience dyspnea for at least 10 min, except for work and location movement?”, (5) “In a week, how many days do you usually perform physical activities of light-to-moderate intensity as mentioned above”, (6) “How long do you usually perform physical activities of light-to-moderate intensity as mentioned above?”. Examples of vigorous intensity include running, jumping rope, hiking, basketball games, swimming, and badminton. Examples of light-to-moderate intensity exercises include jogging, fast walking, weight training, golf, dance sports, and pilates. The total amount of Moderate-Vigorous Physical Activity MVPA (min/wk) was calculated by multiplying the frequency and duration of the session. The total (weighted) MVPA was obtained by calculating the sum of the light-to-moderate intensity duration in minutes and vigorous intensity multiplied by 2. The study population was classified according to the individual levels and patterns of physical activity. Participants were classified into two groups: physical activity (MVPA ≥ 150 min/week) and inactivity (MVPA < 150 min/week) according to the WHO guidelines for physical activity and sedentary behavior [10]. Physical activity groups were further classified according to the frequency of MVPA sessions per week: weekend warriors (≤2 sessions/week) or regularly active (≥3 sessions/week) (Figure 1).

Metabolic syndrome was used as the dependent variable. Its definition was provided by the Third Report of the NCEP Expert Panel on Detection, Evaluation, and Treatment of High Blood Cholesterol in Adults (Adult Treatment Panel III) [17]. It was used to determine metabolic syndrome and its composition and the specific waist circumference (WC) values provided by the WHO and the Korea Obesity Society. These five components were: (1) abdominal obesity (WC ≥ 90 cm in males and ≥ 85 cm in females), (2) high blood pressure (systolic ≥ 130 mmHg or diastolic ≥85 mmHg), (3) low high-density lipoprotein cholesterol level (<40 mmHg/dL in males and <50 mm/dL in females), (4) high-triglyceride level (≥150 mg/dL), and (5) high-glucose level (≥100 mg/dL). Of the five components, three or more participants were classified as a group with metabolic syndrome, which is a cut-off that was widely used [17]. Such components were collected via standardized physical examination by medical technicians serving in the survey.

We controlled for covariates such as sociodemographic characteristics (sex and age), socioeconomic status (marital status, educational level, household income, region, occupation), and health behaviors (smoking status and drinking status) of the participants.

### 2.4. Statistical Analysis

Independent variables were compared using the chi-square test to identify the association of the “weekend warrior” attitude and other physical activity patterns with metabolic syndrome. After adjusting for covariate variables, multiple logistic regression analysis was performed to evaluate the association between physical activity patterns and metabolic syndrome. Moreover, we performed a subgroup analysis stratified by metabolic syndrome and physical activity pattern variables. Furthermore, multiple logistic regression analysis was used to examine the association of weekend warriors with metabolic syndrome, with regularly active participants as the reference group, except for inactive participants. The results were reported as odds ratios (ORs) and confidence intervals (CIs). Two-sided *p*-values were used to evaluate statistical significance, which was set at *p* < 0.05. SAS version 9.4 (SAS Institute Inc., Cary, NC, USA) was used for all analyses, and all estimates were calculated using sample weighting procedures, strata, and clusters assigned to the study population.

## 3. Results

Table 1 presents the participants’ general characteristics. In the study population, 3609 (13.0%) individuals were regularly active, 594 (2.1%) were weekend warriors, and 23,585 (84.9%) were inactive. Among them, 715 (19.8%) and 155 (26.1%) in the regularly active and weekend warrior groups, respectively, and 6927 (29.5%) in the inactive group had metabolic syndrome.

Table 2 reports the findings of logistic regression analysis for the association of physical activity patterns with metabolic syndrome. Compared to the regularly active group, both the weekend warrior group (OR: 1.29, 95% CI: 1.02–1.65) and inactive group (OR: 1.38, 95% CI: 1.25–1.53) had higher odds of metabolic syndrome, which was statistically significant.

Table 3 reports the subgroup analysis stratified by independent variables, which are physical activity patterns. In males, the association with metabolic syndrome increased only in the inactive group (OR: 1.41, 95% CI: 1.24–1.60). However, in females, both the weekend warrior group (OR: 1.70, 95% CI: 1.00–2.89) and the inactive group (OR: 1.43, 95% CI: 1.20–1.70) showed a higher presence. Additionally, the strong association between women’s weekend warrior group (OR: 1.70, 95% CI: 1.00–2.89) and metabolic syndrome indicate the sex-dependent effect of heterogeneity.

**Figure 2:**
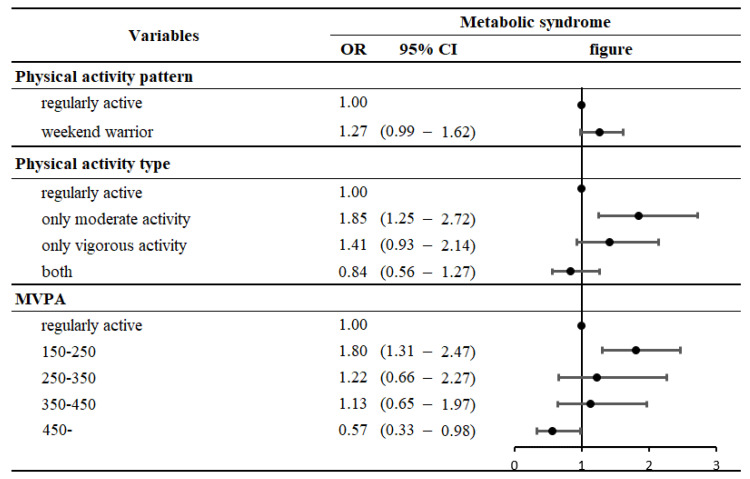
Association of weekend warrior physical activity pattern with metabolic syndrome, with regularly active participants as the reference category.

Figure 2 presents the logistic regression analysis results comparing the weekend warrior and regularly active groups, excluding those with MVPA < 150 according to the WHO guidelines for physical activity. Excluding the inactive group, the weekend warrior group had a higher OR for metabolic syndrome than the regular active group; however, the difference was not statistically significant. Additionally, upon dividing the types of physical activity among the weekend warriors, people who only engaged in moderate-intensity physical activity (OR:1.29, 95% CI:1.02–1.65) and those with an MVPA between 150 and 250 (OR:1.80, 95% CI:1.31–2.47) showed a strong association with metabolic syndrome compared to the regularly active group.

Table 4 shows the subgroup analysis stratified by the dependent variable, which is metabolic syndrome. The inactive group for physical activity was considerably associated with a decreased risk of high-density lipoprotein cholesterol level (OR:1.36, 95% CI:1.23–1.49) and an increased risk of abdominal obesity (OR:1.21, 95% CI:1.10–1.34), triglyceride levels (OR:1.24, 95% CI:1.13–1.37), and glucose levels (OR:1.18, 95% CI:1.08–1.30). In particular, subjects with triglyceride levels were also more likely to have metabolic syndrome among weekend warriors (OR:1.26, 95% CI:1.01–1.58).

## 4. Discussion

The aim of this study was not only to study physical activity but also physical activity patterns such as frequency and intensity with metabolic syndrome. We researched whether the physical activity patterns of the “weekend warrior” group were associated with metabolic syndrome. We found a higher risk of metabolic syndrome in participants in the inactive group and the weekend warriors than in the regularly active group. In addition, previous studies with KNHANES support the same results, based on the mechanism that the risk of metabolic syndrome increases proportionally with age because metabolic syndrome is affected by the aging process [23,24]. People with low-education levels or household incomes are more likely to share housework and livelihood work, leading to increased metabolic syndrome due to stress and economic deprivation [25,26,27]. This burden of household maintenance, irregular dietary habits, and frequent alcohol drinking during social working life are more common among men than women in Korea [28].

However, when only participants with MVPA ≥ 150 (i.e., people who engaged in physical activity) were compared, we did not observe a statistically significant association between the regularly active group and the weekend warriors. This finding suggests there is no significant effect on metabolic syndrome, whether the subjects performed regular or intense physical activities. However, for participants in the weekend warrior category, only subjects with moderate-intensity physical activity or MVPA less than 250 showed an association with metabolic syndrome compared with the regularly active group. Additionally, depending on the type of physical activity and MVPA, there was a dose-dependent tendency between the regularly active group and weekend warriors. In other words, risk has a dose-dependent effect on physical activity type and MVPA. Therefore, we recommend that participants in the weekend warrior category try to achieve high MVPA and perform not only moderate-intensity physical activity but also vigorous-intensity physical activity.

This study supports the results of previous studies showing that inactive individuals are more likely to have metabolic syndrome than active people [10,29,30,31,32,33]. Similarly, Framingham Heart Study revealed a relationship between physical activity guidelines and several cardiac metabolic risk factors using accelerometer data cross-sectional analysis [31]. The authors found that those who met the total MVPA guidelines had lower WC and triglyceride levels and higher HDL cholesterol [31]. Although previous physical activity guidelines recommend that adults regularly accumulate at least 30 min of MVPA for a week [34], current guidelines do not impose any recommendations on the frequency of MVPA throughout the week, as research on weekend warriors is scarce [10,29]. Therefore, our results support the notion that the frequency of MVPA throughout the week is not as important as the total amount of MVPA [35]. Our findings suggest that guidelines are needed to help people increase their total MVPA and perform vigorous-intensity physical activity.

A previous study on the relationship between metabolic syndrome and related diseases and physical activity patterns among the China rural adults was studied [36]. Our study included a group that insufficient active group as an inactive group, but in a previous study, they studied them separately. It is also a cross-sectional study of rural adults over a short period of one year. Due to these differences, our study results may be slightly different, but the conclusion is that the “weekend warrior” who engaged in moderate to vigorous physical activity for high intensity was associated with a reduced incidence of metabolic syndrome [36]. In addition, this study focused on diseases such as hypertension and diabetes associated with metabolic syndrome [36]. In support of this study, the mechanism between physical activity and metabolic syndrome is also associated with obesity followed by hypertension and diabetes [36,37,38]. There are mechanisms by which physical activity reverses diabetes-related muscle atrophy, improving metabolic function, and reducing inflammation, oxidative stress, and mitochondrial damage [37]. Moreover, some studies have found that regular physical activity patterns control cholesterol levels and blood pressure to manage weight [38]. Furthermore, it has been reported that physical activity is already a proven way to manage type 2 diabetes and hypertension, which has the potential to lead to the management of metabolic syndrome as well [38].

This study had certain limitations. First, it used data from a cross-sectional study. While our study showed an association between the “weekend warrior” attitude and other physical activity patterns with metabolic syndrome, our results should be interpreted cautiously because it could be an inverse causal relationship. Therefore, further longitudinal studies are needed to clarify the relationship between physical activity patterns and metabolic syndrome. Second, the KNHANES was collected as a self-report survey. Physical activity as our variable of interest was evaluated using data prone to measurement errors without quantitative measurement tools, such as accelerometers. Although the KNHANES did not have tools to evaluate physical activity, it included all kinds of information about the frequency, duration, and type of physical activity obtained through six questionnaires. Other studies have used this method to minimize bias by using an overall reliable MVPA calculation formula. Therefore, future studies should include both self-reporting and device measurements of physical activity. Third, we recognized that the standard of physical activity was only leisure time, excluding work and locomotion. Therefore, different results may be reported for people who perform extensive physical activity during work and locomotion. Fourth, measurements of acute physiological and metabolic changes that persist after physical activity remain unclear [39,40,41,42]. Particularly, HDL-cholesterol can improve from 24 h to 72 h, and improvement in insulin sensitivity can last several days [39,40,41,42]. Therefore, future studies should consider the timing of cardiac metabolism and physical activity measurements and be asked not to exercise for 1 day before collecting individual blood samples. Finally, while we adjusted for known confounding factors affecting the relationship between physical activity and metabolic syndrome, we cannot rule out the influence of residual confounding factors such as dietary intake. Since there is a large association between dietary intake and metabolic syndrome, we recommend that future research is used accurate dietary intake data and included it as a confounding variable.

Nonetheless, this study had several strengths. First, this study was based on the KNHANES, the most recent nationally representative data that can generalize the research results to the general adult population in Korea. This is reliable and is performed using a special random cluster sampling. Second, to compare weekend warriors and regularly active groups in more detail, we looked closely at the types of physical activity and MVPA of the participants. These findings encourage weekend warriors to manage their physical activity and time more effectively and help prevent metabolic syndrome.

## 5. Conclusions

In conclusion, our study emphasizes that the weekend warrior group was more likely to develop metabolic syndrome than the regularly active groups, and encourage them to do vigorous as well as moderate intensity physical activity so that MVPA is high. Therefore, for those with fewer opportunities to engage in regular physical activity on weekdays, physical activities of both moderate and vigorous intensity contribute greatly to the health of the weekend warrior group. We suggest that weekend warrior physical activity patterns should be reflected in future physical activity guidelines and interventions.

## Figures and Tables

**Table 1:** General characteristics of the study population.

Variables		Metabolic Syndrome						
		Total		Yes		No		*p*–Value
		N	%	N	%	N	%	
Total		27,788	100.0	7837	28.2	19,951	71.8	
Physical activity patterns								<0.0001
	Regularly active	3609	13.0	715	19.8	2894	80.2	
	Weekend warrior	594	2.1	155	26.1	439	73.9	
	Inactive	23,585	84.9	6967	29.5	16,618	70.5	
Sex								<0.0001
	Male	12,302	44.3	4008	32.6	8294	67.4	
	Female	15,486	55.7	3829	24.7	11,657	75.3	
Age								<0.0001
	19–29	3504	12.6	289	8.2	3215	91.8	
	30–39	4240	15.3	750	17.7	3490	82.3	
	40–49	5089	18.3	1256	24.7	3833	75.3	
	50–59	5313	19.1	1622	30.5	3691	69.5	
	60–69	5001	18.0	1914	38.3	3087	61.7	
	70–	4641	16.7	2006	43.2	2635	56.8	
Marital status								<0.0001
	Married	19,029	68.5	5602	29.4	13,427	70.6	
	Single, widow	7311	26.3	1762	24.1	5549	75.9	
	Divorced, Separated	1448	5.2	473	32.7	975	67.3	
Educational level								<0.0001
	Middle school or below	8038	28.9	3335	41.5	4703	58.5	
	High school	9186	33.1	2325	25.3	6861	74.7	
	College or over	10,564	38.0	2177	20.6	8387	79.4	
Household income								<0.0001
	Low	5105	18.4	1995	39.1	3110	60.9	
	Mid–low	6750	24.3	2023	30.0	4727	70.0	
	Mid–high	7633	27.5	1943	25.5	5690	74.5	
	High	8300	29.9	1876	22.6	6424	77.4	
Region								<0.0001
	Metropolitan	12,275	44.2	3228	26.3	9047	73.7	
	Urban	10,294	37.0	2828	27.5	7466	72.5	
	Rural	5219	18.8	1781	34.1	3438	65.9	
Occupational categories								<0.0001
	White	7027	25.3	1515	21.6	5512	78.4	
	Pink	3637	13.1	927	25.5	2710	74.5	
	Blue	6333	22.8	2106	33.3	4227	66.7	
	Inoccupation	10,791	38.8	3289	30.5	7502	69.5	
Current smoking status								<0.0001
	Non–smoker	16,676	60.0	4208	25.2	12,468	74.8	
	Ex–smoker	6163	22.2	2002	32.5	4161	67.5	
	Current–smoker	4949	17.8	1627	32.9	3322	67.1	
Current drinking status								<0.0001
	Never or occasionally	7621	27.4	2532	33.2	5089	66.8	
	2~4 times/month	14,105	50.8	3371	23.9	10,734	76.1	
	2~4 times/week	6062	21.8	1934	31.9	4128	68.1	
Year								0.0055
	2016	5558	20.0	1571	28.3	3987	71.7	
	2017	5549	20.0	1474	26.6	4075	73.4	
	2018	5724	20.6	1594	27.8	4130	72.2	
	2019	5740	20.7	1645	28.7	4095	71.3	
	2020	5217	18.8	1553	29.8	3664	70.2	

**Table 2:** Results of factors associated between physical activity patterns and metabolic syndrome.

Variables		Metabolic Syndrome			
		OR	95% CI		
Physical activity patterns					
	Regularly active	1.00			
	Weekend warrior	1.29	(1.02	–	1.65)
	Inactive	1.38	(1.25	–	1.53)
Sex					
	male	1.90	(1.73	–	2.08)
	female	1.00			
Age					
	19–29	1.00			
	30–39	2.88	(2.40	–	3.45)
	40–49	4.37	(3.64	–	5.25)
	50–59	5.60	(4.65	–	6.74)
	60–69	6.19	(5.11	–	7.49)
	70–	6.81	(5.62	–	8.25)
Marital status					
	Married	1.00			
	Single, widow	1.21	(1.10	–	1.33)
	Divorced, Separated	1.00	(0.87	–	1.15)
Educational level					
	Middle school or below	1.62	(1.45	–	1.81)
	High school	1.19	(1.08	–	1.30)
	College or over	1.00			
Household income					
	Low	1.00			
	Mid–low	0.91	(0.82	–	1.01)
	Mid–high	0.86	(0.77	–	0.96)
	High	0.81	(0.72	–	0.91)
Region					
	Metropolitan	1.00			
	Urban	1.06	(0.98	–	1.14)
	Rural	1.12	(1.02	–	1.24)
Occupational categories					
	White	1.00	(0.91	–	1.11)
	Pink	1.00	(0.90	–	1.12)
	Blue	0.85	(0.77	–	0.93)
	Inoccupation	1.00			
Current smoking status					
	Non–smoker	1.00			
	Ex–smoker	0.99	(0.89	–	1.09)
	Current–smoker	1.21	(1.09	–	1.35)
Current drinking status					
	Never or occasionally	1.00			
	2~4 times/month	0.89	(0.82	–	0.97)
	2~4 times/week	1.06	(0.95	–	1.17)
Year					
	2016	1.00			
	2017	0.89	(0.79	–	1.00)
	2018	0.98	(0.87	–	1.09)
	2019	1.06	(0.95	–	1.19)
	2020	1.09	(0.98	–	1.22)

**Table 3:** Results of subgroup analysis stratified by independent variables.

		Metabolic Syndrome								
		Regularly Active	Weekend Warrior				Inactive			
		OR	OR	95% CI			OR	95% CI		
Sex										
	male	1.00	1.14	(0.87	–	1.50)	1.41	(1.24	–	1.60)
	female	1.00	1.70	(1.00	–	2.89)	1.43	(1.20	–	1.70)
Age										
	19–29	1.00	1.29	(0.62	–	2.70)	1.38	(0.97	–	1.96)
	30–39	1.00	1.14	(0.64	–	2.02)	1.07	(0.81	–	1.41)
	40–49	1.00	1.11	(0.62	–	1.98)	1.81	(1.41	–	2.32)
	50–59	1.00	1.49	(0.94	–	2.36)	1.58	(1.26	–	1.98)
	60–69	1.00	1.45	(0.81	–	2.58)	1.33	(1.08	–	1.64)
	70 –	1.00	0.99	(0.32	–	3.03)	1.00	(0.73	–	1.35)
Marital status										
	Married	1.00	1.35	(1.02	–	1.79)	1.38	(1.22	–	1.56)
	Single, widow	1.00	1.10	(0.64	–	1.89)	1.31	(1.04	–	1.67)
	Divorced, Separated	1.00	1.19	(0.37	–	3.86)	1.96	(1.14	–	3.36)
Household income										
	Low	1.00	3.78	(1.24	–	11.52)	1.40	(1.03	–	1.91)
	Mid–low	1.00	0.77	(0.45	–	1.30)	1.25	(1.00	–	1.56)
	Mid–high	1.00	1.45	(0.92	–	2.27)	1.23	(1.01	–	1.49)
	High	1.00	1.27	(0.89	–	1.82)	1.63	(1.38	–	1.93)
Occupational categories										
	White	1.00	1.24	(0.83	–	1.87)	1.46	(1.20	–	1.76)
	Pink	1.00	1.29	(0.66	–	2.51)	1.30	(0.97	–	1.75)
	Blue	1.00	1.52	(0.94	–	2.45)	1.44	(1.13	–	1.85)
	Inoccupation	1.00	1.07	(0.61	–	1.87)	1.38	(1.16	–	1.63)

**Table 4:** Results of subgroup analysis stratified by dependent variables.

		Physical Activity Patterns								
		Regularly Active	Weekend Warrior				Inactive			
		OR	OR	95% CI			OR	95% CI		
Metabolic syndrome components	Abdominal obesity	1.00	0.96	(0.76	–	1.21)	1.21	(1.10	–	1.34)
	High BP	1.00	0.99	(0.79	–	1.23)	0.99	(0.90	–	1.09)
	Low HDL	1.00	1.24	(0.99	–	1.55)	1.36	(1.23	–	1.49)
	High TG	1.00	1.26	(1.01	–	1.58)	1.24	(1.13	–	1.37)
	High Glucose	1.00	1.06	(0.85	–	1.32)	1.18	(1.08	–	1.30)

## Data Availability

The dataset analyzed in this study is publicly accessible (https://www.kdca.go.kr (accessed on 12 August 2022)).
